# Monitoring river water and sediments within a changing Ethiopian catchment to support sustainable development

**DOI:** 10.1007/s10661-019-7545-6

**Published:** 2019-06-21

**Authors:** E. Zinabu, P. Kelderman, J. van der Kwast, K. Irvine

**Affiliations:** 1 IHE Delft, P.O. Box 3015, 2601 DA Delft, The Netherlands; 2 Wollo University, P.O. Box 1145, Dessie, Ethiopia; 3 Aquatic Ecology and Water Quality Management, Wageningen University, P.O. Box 47, 6700AA, Wageningen, The Netherlands

**Keywords:** Ethiopia, Metals, Monitoring, River, Policy, Pollution

## Abstract

In many sub-Saharan states, despite governments’ awareness campaigns highlighting potential impacts of aquatic pollution, there is a very limited action to protect the riverine systems. Managing the quality of water and sediments needs knowledge of pollutants, agreed standards, and relevant policy framework supporting monitoring and regulation. This study reports metal concentrations in rivers in industrializing Ethiopia. The study also highlights policy and capacity gaps in monitoring of river and sediments. For two sampling periods in 2013 and 2014, chromium (Cr), copper (Cu), zinc (Zn), and lead (Pb) were monitored in water and sediments of the Leyole and Worka rivers in the Kombolcha city, Ethiopia. The sampling results were compared with international guidelines and evaluated against the Ethiopian water protection policies. Chromium was high in the Leyole river water (median 2660 μg/L) and sediments (maximum 740 mg/kg), Cu concentrations in the river water was highest at the midstream part of the Leyole river (median 63 μg/L), but maximum sediment content of 417 mg/kg was found further upstream. Zinc was the highest in the upstream part of the Leyole river water (median 521 μg/L) and sediments (maximum 36,600 mg/kg). Pb concentrations were low in both rivers. For the sediments, relatively higher Pb concentrations (maximum 3640 mg/kg) were found in the upstream of the Leyole river. Except for Pb, the concentrations of all metals surpassed the guidelines for aquatic life, human, livestock, and irrigation water supplies. The median concentrations of all metals exceeded guidelines for sediment quality for aquatic organisms. In Ethiopia, poor technical and financial capabilities restrict monitoring of rivers and sediments and understanding on the effects of pollutants. The guidelines used to protect water quality is based on the World Health Organization standards for drinking water quality, but this is not designed for monitoring ecological health. Further development of water quality standards and locally relevant monitoring framework are needed. Development of monitoring protocols and institutional capacities are important to overcome the policy gaps and support the government’s ambition in increasing industrialization and agricultural intensification. Failure to do so presents high risks for the public and the river ecosystem.

## Introduction

Pollution of surface waters is a ubiquitous problem especially in the developing countries where expansion of industry and intensification of agriculture are not matched with suitable water quality policies or, more commonly, their enforcement. In these countries, monitoring infrastructures are poor and often lack management and scientific capacity (Hove et al. 2013; Commission 2011). Economic and financial pressures dominate other concerns and the impact of pollutants on water is neglected (Abbaspour 2011).

Because of their toxicity and persistence in aquatic systems, metal pollution is a concern for public and ecosystem health (Yuan et al. 2011; Armitage et al. 2007). Metals from industry and agricultural sources often end up in rivers and sediments (Islam et al. 2014; Su et al. 2013), and these metals are usually subjected to remobilization driven by changes in hydrology, organic matter, and sediment grain size (Kelderman 2012; Yi et al. 2011). In sub-Saharan Africa, while many of the countries have adopted environmental quality standards, monitoring of metals in river water and sediment for legal compliance is hardly conducted (Chikanda 2009). Ethiopia is among these countries that provides an example of high pollution risks from industrial and agricultural activities that has remained unknown.

Ethiopia has endorsed several international conventions and agreements for water protection (EEPA 2010). The country is a signatory to the Sustainable Development Goals (SDGs) and has aligned its second Growth and Transformation Plan (GTP-II) to the sustainable development (FDRE 2016). Despite the Ethiopian government’s awareness about impacts of pollution, there is limited action to protect river ecosystem health (Akele et al. 2016; Aschale et al. 2016; Beyene et al. 2009). Rapid urbanization and industrialization continue to degrade surface waters (EEPA 2010; FDRE 2002). Policies regarding water protection are limited to the regulation of pollutant emission into waters through a “polluter pays” principle and no preventive management has been implemented to protect pollutant-receiving rivers.

The city of Kombolcha, in north-central Ethiopia, is a typical sub-Saharan conurbation with high pollutant emissions from industrial and municipal discharges and agricultural intensification (Zinabu et al. 2017a). While it is apparent that some stretches of the rivers running through the city are polluted, information on water and sediment quality of the rivers is scant. Some of the rivers receive discharges from multiple industrial sources and heavy metals like Cu, Zn, and Pb are suspected to be released by the local steel product processing factories, while Cr is expected to be discharged by the local tannery. The local brewery and meat processing factory are also supposed to discharge organic wastes and influence the distribution of these metals in the effluent-receiving rivers. Most of the factories’ effluents are not monitored, although few factories are monitoring their effluent discharges only for BOD, COD, total suspended solids, and pH (Zinabu et al. 2017a). In Ethiopia, only the WHO drinking-water quality guidelines are used to evaluate surface water quality and no national or local environmental standards are applied to protect the river ecosystem (EMoWIE 2016). In this study, concentrations of the metals chromium (Cr), zinc (Zn), copper (Cu), and lead (Pb) were monitored over a 2-year period in two rivers of Kombolcha city, and the metal concentrations were compared with “a compendium of environmental quality benchmarks” (MacDonald et al. 2000a) and sediment quality standards that are described in “the numerical Sediment Quality Guidelines (SQGs)” (MacDonald et al. 2000a; USEPA 1997a). Information from local government and review of policies in Ethiopia and the sub-Saharan regions were used to present a more general overview of (i) application of standards, (ii) compliance, and (iii) recommendation for improvement.

## Materials and methods

### Study area description

**Fig. 1:**
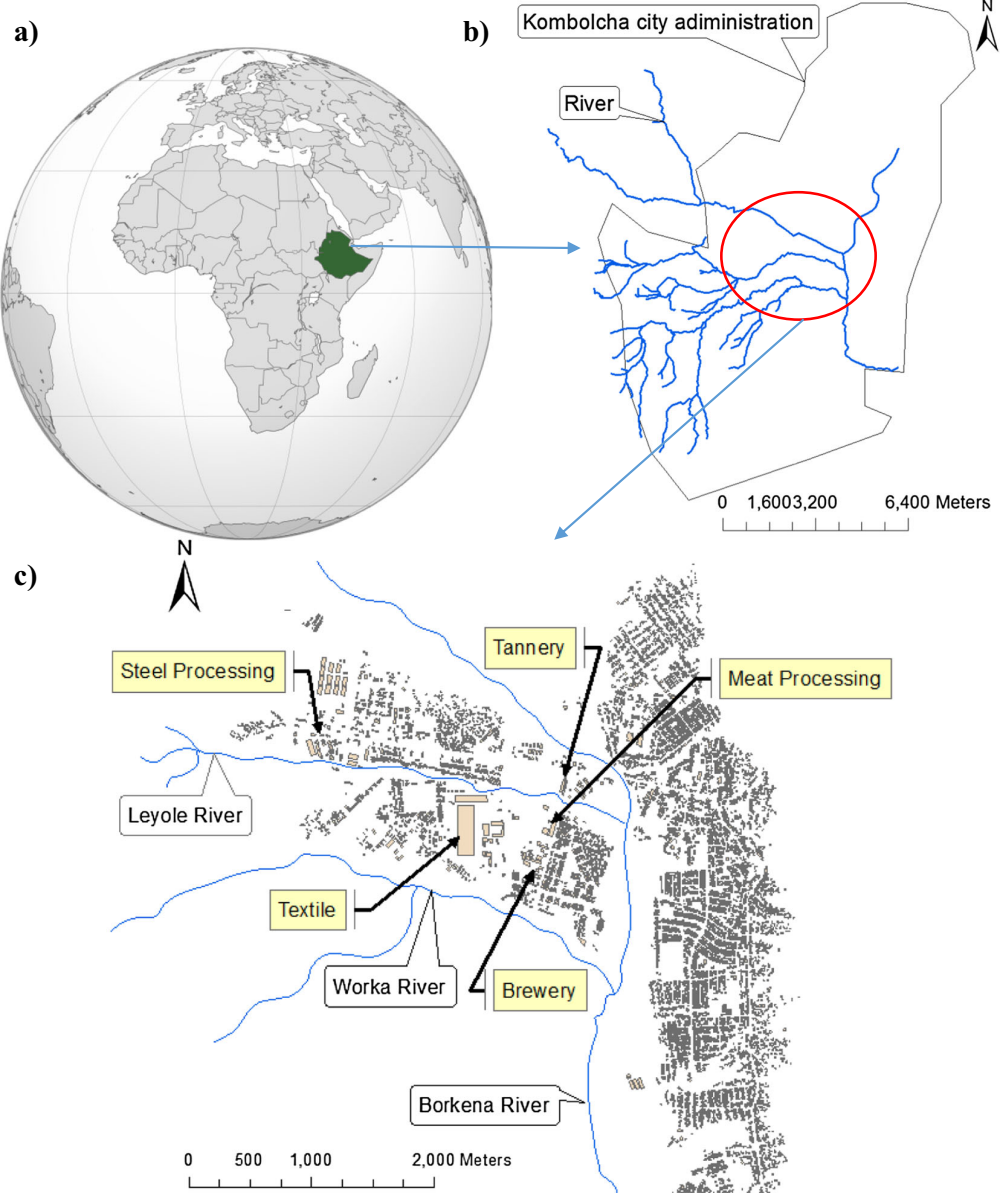
The location of the study area on the horn of Africa (**a**), in the Kombolcha city administration (**b**), and within the industrial zone areas and codes (**c**): LD1 (confluence of three upstream tributaries and start of upstream Leyole river); LD2 (downstream of effluent discharge of steel processing factory in the Leyole river); LD3 (downstream of effluent discharge of textile in the Leyole river); LD4 (downstream of tannery effluent discharge in the Leyole river); LD5 (downstream of meat processing effluent discharge in the Leyole river); WD1(upstream Worka river); and WD2 (downstream of brewery effluent discharge in the Worka river)

The Kombolcha administration city is located in the north-central part of Ethiopia (Fig. 1a, b). The administration area covers 125 km^2^ and comprises agricultural and forest land in the rural uplands and peri-urban area, and urbanized and industrial areas in lowland plains. *Vertisol* is the predominant soil type, with *fluvisols* and *cambisol* soil types common along the river bank and in the foothills, respectively (Zinabu 2011). The city has a semi-arid climate with two wet seasons, with the early and relatively short wet season from February to April, and later and heavier rains in the summer from July to September. The city is already one of the major industrial destinations of the country and more industries are envisaged to be established in the near future. This study was conducted in the Leyole and Worka rivers receiving effluents from manufacturing industries including textile, steel product processing, tannery food processing, and brewery (Fig. 1c). These rivers have very low water flows in the dry season and are used for irrigation and water supply and wetland recharges in the downstream (Zinabu 2019). They drain into the Borkena river, which is in turn a tributary of the Awash river (Fig. 1c).

### Sample collection, preservation, and analysis

#### River water monitoring and analysis

Using 100-mL polyethylene (PE) containers, water samples were collected to measure total dissolved Cr, Cu, Zn, and Pb. The samplings were located upstream of the Leyole and Worka rivers (LD1; WD1); downstream of the factories are effluent discharge points in the Leyole river (LD2–5) and Worka river (WD2) (Fig. 1c). The sampling was conducted for two periods in the rainy season starting from June to September, in 2013 (C1) and 2014 (C2). In total, with 16 monitoring dates, 112 water samples were collected from the seven stations. On each sampling occasion, pH and electrical conductivity (EC) were measured in situ using a portable pH meter (WTW, pH340i) and an EC (WTW, cond330i) meter, respectively (Zinabu et al. 2017a).

The water samples were acidified to a pH < 3, by adding 1 mL concentrated H_2_SO_4_, so as to prevent metal adsorption to the container wall. After transporting the samples to the laboratory of IHE-Delft (Institute for Water Education, The Netherlands), they were processed following the ISO 5667-3 procedures (ISO 2003). For processing, 10 mL sample was filtered through Whatman GF-C glass microfiber filters (pore size 1.3 μm), and a final volume was made up to 100 mL using Milli-Q water. Metal concentrations were measured using ICP-MS, XSERIES 2 IUS-MS. All analyses were done in accordance with “Standard Methods” (Rice et al. 2012).

#### Sediment monitoring and processing

Riverbed sediment samples were taken at six stations (LD2–5; WD1–2) on three occasions (Fig. 1): 15 June (M1), 15 July 2013 (M2), and 15 July 2014 (M3) (in total 18 samples). Unlike the sampling of water, sediment samples were not taken from LD1, as we assumed that measurements of metals from this station represent background concentrations from geology and upstream agriculture lands. At each location, the samples were collected with a PE (polyethylene) spoon from the upper 3 cm of the sediment depositions at four evenly spaced points across the width of the rivers. Each sample was mixed, pooled, and stored in 250-mL PE beakers. Then, the resulting sample, approximately 50 g of sediment, was stored in the dark and then transported to the laboratory of IHE-Delft within 4 months. In the laboratory, the samples were air-dried in a dark room within 6 weeks and, subsequently, analyzed within 60 days.

To determine sediment particle size distribution, samples were mixed and homogenized in Milli-Q water added at a 1:1 by volume, followed by stirring of slurry overnight at 150 rpm (IKA RW20) to dissipate clay aggregates. Slurries were then wet-sieved and shaken with warm tap water (Tritsch, mesh size 230 μm) to get particle size fractions of 63–125 μm; 125–500 μm, 500 μm–1 mm, and 1–2 mm. The fraction > 2 mm was not taken as part of “sediment” (Håkanson and Jansson 1983). The grain size fraction < 63 μm in size was estimated by collecting all sediment particles that can pass through the 63-μm sieve after pre-settling overnight in a 25-L PE bucket. We determined the percentages of the five grain size fractions after transferring the sediments into a known weight plastic dishes and drying the samples in an oven-dry at 70 °C until attaining constant weight. We then calculated (a) median grain size (50% larger; 50% smaller) and (b) sorting coefficient (S.C.) 1 (Håkanson and Jansson 1983). The proportion of grain sizes at each station were computed using a probability paper (Boggs 2009). Both median grain size and sorting coefficient are expressed in dimensionless phi units^2^^2^.

To determine organic matter (OM) content, approximately 1 g of each sediment sample was first heated in an oven at 70 °C till getting a constant weight. After this, weight loss was determined by ignition at 520 °C for 3 h (Rice et al. 2012). To measure metal concentrations, triplicates of 0.5 g of the sediment fractions were taken from each grain size fraction and transferred into a Teflon tube and then acid-digested with 10 mL 65% concentrated HNO_3_. The fractions were then dried and digested in a microwave oven (MARS 5) to determine the metal contents (mg/kg) using an inductively coupled plasma mass spectrometer (ICP-MS), Thermo Scientific X Series 2®, followed by dilution with Milli-Q water.

#### Statistical techniques

Since the relative standard deviations of the dataset of each metal were high and the mean values are affected by a few outliers (Table 1), median values of the dataset were used to better represent the metal concentrations at each station (Bartley et al. 2012; Pagano and Gauvreau 2000).

**Table 1:** Estimates of metal concentrations (μg/L) at stations LD1–5 in the Leyole river and WD1–2 in the Worka river during the first (C1) and second campaign (C2), from June–September 2013 and 2014, respectively

Station		LD1		LD2		LD3		LD4		LD5		WD1		WD2	
Monitoring periods (*n* = 8)		C1	C2	C1	C2	C1	C2	C1	C2	C1	C2	C1	C2	C1	C2
pH	Median	7.5	8.0	8.1	8.3	8.3	8	7.8	7.9	7.6	7.6	8.1	8.4	6.3	9.5
	Standard error	0.82	0.13	0.84	0.13	0.89	0.13	0.83	0.07	0.84	0.09	0.83	0.1	0.74	0.58
Cr	Median (μg/L)	4	2	12	6	8	51	9	2660	9	284	2	2	7	38
	Mean (μg/L)	3	437	11	380	7	230	9	6880	11	4280	3	37	8	30
	Maximum (μg/L)	21	2690	44	2160	25	1130	15	25,900	16	18,250	5	154	13	73
	Minimum (μg/L)	2	1	2	0.7	2	0.7	2	206	2	26	2	1	2	2
	Standard error	4	330	5	260	3	140	9	3360	6	2580	0	22	1	9
Cu	Median (μg/L)	23	0.4	17	14	63	41	10	21	14	27	8	0.2	13	33
	Mean (μg/L)	80	305	83	268	101	155	41	85	65	188	51	34	73	354
	Maximum (μg/L)	303	1900	248	1540	254	827	254	358	268	1180	270	154	274	2450
	Minimum (μg/L)	3	0.1	7	0.1	4	0.1	3	0.1	3	0.1	2	0.1	3	0.1
	Standard error	37	237	36	191	37	101	30	45	33	144	33	22	35	301
Zn	Median (μg/L)	72	110	95	521	71	187	30	205	81	214	41	137	106	194
	Mean (μg/L)	77	110	109	886	91	525	52	384	127	528	67	151	194	175
	Maximum (μg/L)	126	3310	367	2780	218	1600	131	1050	611	2120	143	338	855	278
	Minimum (μg/L)	26	16	29	9	54	34	15	67	15	25	9	12	14	46
	Standard error	15	402	37	365	21	209	17	127	65	250	19	45	92	29
Pb	Median (μg/L)	2	1	3	1	3	3	4	5	3	0.8	2	1	4	1
	Mean (μg/L)	1	11	1	10	1	8	0.4	128	1	8	3	2	2	1
	Maximum (μg/L)	5	70	6	60	5	34	4	980	4	44	4	7	5	5
	Minimum (μg/L)	2	1	2	1	2	1	2	0.6	2	0.6	2	1	2	1
	Standard error	0.4	8	0.7	7	0.4	4	4	121	0.4	5	0.3	0.7	0.2	0.4

Two-way repeated measurements ANOVA (analysis of variance) was used to test temporal differences of metal concentrations within and between the two sampling seasons. Analysis was done in R statistical package using “ANOVA” in a “CAR” (companion to applied regression) statistical package (Fox and Weisberg 2011; R Core Team 2015). As the dataset is not normally distributed, a statistically significant difference between tested times frames was estimated by Kruskal-Wallis one-way analysis of variance at *р* ≤ 0.05, and Tukey’s HSD multiple comparisons.

#### Quality assurance

All the reagents used were of analytical grade, and sample dilutions were made with Milli-Q water. The quantification of metal concentrations was based on calibration curves of standard solutions for the metals. Detection limits were as follows: Cr: < 0.07 μg/L; Cu :< 0.01 μg/L; Zn: < 0.1 μg/L; Pb: < 0.06 μg/L. The precision of the analytical procedures, which is expressed as the relative standard deviation (RSD), was 5% – 10%. The ICP-MS measurements always had an RSD < 5% in the laboratory analyses. In all experiments, blanks were run; and during digestion of the sediment samples, two blanks of 65% HNO_3_ and two standards of certified reference materials (sewage sludge-amended soil No. 143 R ID 0827) were used. The calculated recovery percentages were 100%, 103%, 104%, and 105 %, for Cr, Cu, Zn, and Pb, respectively.

Water hardness, which is measured as the concentration of CaCO_3_ (mg/l) in the water, affects bioavailability of metals in rivers (Besser et al. 2001; Pourkhabbaz et al. 2011). For the rivers of Kombolcha, a previous study showed that the hardness of the Leyole and Worka rivers water is < 60 mg/l and classified the river waters as “soft hardness water” (Zinabu 2011). We, therefore, evaluated the sampling results under the conditions of “soft” water in comparing the metal concentrations compliance with guidelines for the protection of aquatic lives, human, livestock, and irrigation water supplies that are outlined in “a compendium of environmental quality benchmarks” (MacDonald et al. 2000a).

To examine the environmental quality of sediments in the rivers, we used the numerical Sediment Quality Guidelines (SQGs) (USEPA 1997a; MacDonald et al. 2000b). The sediment quality of each sample taken from the stations was compared with Threshold Effect Concentration (TEC) and a Probable Effect Concentration (PEC) guidelines. TECs are the contents below which adverse effects on sediment-dwelling organisms are not expected, while PECs are the contents above which adverse effects are expected to occur frequently, and which may call for urgent remedial actions (Swartz 1999; MacDonald et al. 2000b).

## Results

### Metals in the Leyole and Worka rivers

#### Comparisons with water quality guidelines

**Fig. 2:**
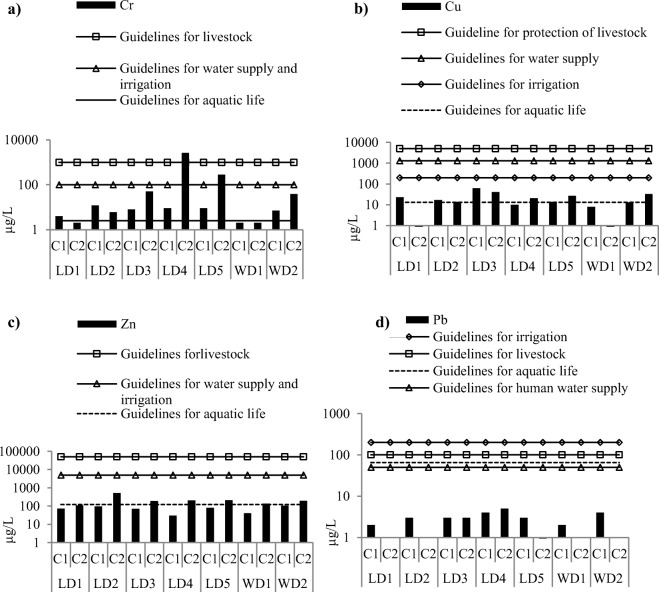
Median metal concentrations (μg/L) at the monitoring stations (see Fig. 1c) of the Leyole river and Worka river for the 2013 (C1) and 2014 (C2) monitoring periods; also the different water quality guidelines are presented (Guidelines for protection of aquatic life in μg/L (for hardness ≤ 100 mg/L): Cr (2.5), Cu (13), Zn (120), Pb (65) (USEPA 1998). Guidelines for protection of human health (WHO/UNICEF 2014) in μg/L, for Cr(100), Cu (1300), and Pb(50), for Zn (5000) (USEPA 1986). Guidelines for protection of irrigation in μg/L, for Cr(100), (Nagpal et al. 1995); for Cu(200), Zn(5000); ( soil pH > 6.5) and Pb (200) (CCREM 2001). Guideline for protection of livestock in μg/L, for Cr(1000) (Nagpal et al. 1995); for Cu(5000), Zn(50000), and Pb(100) (CCREM 2001))

Except for Pb, all metal concentrations exceeded one or more water quality guidelines (Fig. 2). As expected, the highest median concentrations of total dissolved Cr were found downstream of the tannery outflow at station LD4 in the Leyole river (Table 1). At this station, the mean concentration of Cr exceeded all of the guideline limits and the maximum concentrations (25.9 mg/L) was recorded in 2014 (Fig. 2a). Although median Cr concentrations decreased at LD5, the concentration in the river water still exceeded the guideline limit for protection of livestock water supply. At the other sampling stations, median Cr concentrations did not exceed all of the guidelines. However, maximum Cr concentrations of some of these stations have exceeded especially for the monitoring period C2 (i.e. in 2014). In the Worka river, at stations WD1 and WD2, Cr concentrations were relatively low compared with the Leyole river. The Cr guideline limit for protection of aquatic life was yet exceeded at these stations (Fig. 2a). For both monitoring periods, median concentrations of dissolved Cu were close to or exceeded the guideline limits for protection of aquatic life at all stations of the Leyole and Worka rivers (Fig.2b). In 2013, the highest median record (0.06 mg/L) was found at LD3 (i.e., in the textile effluent mix in the Leyole river) (Table 1). A similar pattern was observed for Zn (Fig.2c), with the highest median concentration at LD2 in the steel processing effluent outflow. The highest maximum concentration of Pb (2.45 mg/L) was found downstream of the brewery outflow in the Worka river (Table 1), but the Pb concentrations were often well below all water quality guideline limits (Fig. 2d).

Finally, contrary to expectation, high median Cu and Zn concentrations were recorded at station LD1 compared to the stations in the downstream parts, upstream of the industrial discharges in the Leyole river (Fig. 2a, b), possibly related to solid wastes of the factories in the vicinity (see later discussion for Zn).

While comparing the metal concentration among the stations, significantly different (*p* ≤ 0.05, the Kruskal-Wallis test) concentrations of Cr were found between stations LD1 and LD4 (using Tukey’s HSD multiple comparisons test, *p* = 0.03) (Table 2). No significant difference in the concentration between stations was found for the other three metal concentrations (Table 2). Similarly, significant differences in temporal patterns (i.e., within each sampling period and between sampling periods of C1 and C1) were found for Cu and Zn concentrations in the Leyole river (respectively, *p* ≤ 0.05, 0.01) (using repeated-measures ANOVA) (Table 3). In the Worka river, statistically significant differences were found only for Cr between the sampling periods, and for Pb within the periods.

**Table 2:** Univariate Type III Repeated-Measures ANOVA test result for biweekly (BW) and monitoring period (MP) levels of mean metals concentrations monitored at stations LD1-5 in the Leyole river and WD1-2 in the Worka river

River	Metals	Level of test	SS numa	d.f.^b^	Error SS^c^	den d.f.^d^	F^e^	Pr (> *F*)^f^
Leyole river	Cr	MP	1.18E+08	1	1.45E+08	4	3.2	0.145
		BW	1.24E+08	7	3.84E+08	28	1.2	0.292
	Cu	MP	3.12E+05	1	1.01E+05	4	12	0.024*
		BW	3.19E+06	7	1.42E+06	28	9.0	8.532e−06***
	Zn	MP	5.11E+06	1	5.31E+05	4	38	0.003**
		BW	7.20E+06	7	4.97E+06	28	5.7	0.000***
	Pb	MP	7.96E+04	7	3.39E+05	28	0.9	0.492
		BW	1.87E+04	1	4.56E+04	4	1.6	0.270
Worka river	Cr	MP	5.17E+03	1	3.00E+00	1	1721	0.015*
		BW	7.51E+03	7	4.42E+03	7	1.7	0.250
	Cu	MP	8.95E+04	1	2.24E+05	1	0.4	0.641
		BW	1.13E+06	7	1.32E+06	7	0.8	0.577
	Zn	MP	1.86E+02	1	1.27E+04	1	0.01	0.923
		BW	1.97E+05	7	2.05E+05	7	0.9	0.518
	Pb	MP	6.28E+00	1	2.33E+00	1	2.7	0.348
		BW	3.80E+01	7	6.70E+00	7	5.7	0.017*

^a^Sum of squares for numerator; ^b^degree of freedom, ^c^error sum of square, ^d^denumerator degree of freedom, ^e^*F* values, ^f^*p* values: *** 0.001; ** 0.01; * 0.05

**Table 3:** Kruskal-Wallis rank sum test of metals among sampling stations along the Leyole river; d.f., degrees of freedom; and Tukey’s HSD test for significantly varied metal (*p* adj. = 0.05)

Test group (LD1, LD2, LD3, LD4, LD5)	d.f.^a^	Kruskal-Wallis chi-squared	*p* values
Cr	4	13	0.01*
Cu	4	1.2	0.88
Zn	4	2	0.82
Pb	4	1	0.89
Test metal for HSD	Stations compared	p adj.	
Cr	LD1 vs. LD2	0.9	
	LD1vs. LD3	0.9	
	LD1 vs. LD4	0.03*	
	LD1 vs. LD5	0.07	
	LD2 vs. LD3	1	
	LD2 vs. LD4	0.21	
	LD2 vs. LD5	0.38	
	LD3 vs. LD4	0.24	
	LD3 vs. LD5	0.42	
	LD4 vs. LD5	1	

*d.f.* degree of freedom

*Significant at *p* ≤ 0.05

### Physicochemical characteristics and metal concentrations of sediments in the Leyole and Worka rivers

Detailed grain size analyses are presented in Fig 4b. The grain size distributions of the sediment samples were variable across the sampling stations in the Leyole and Worka rivers. Coarse sand (< 1.0 phi units) were found at LD3, LD5, and WD2, while the other stations were dominated by fine to very fine sand between 2.0 and 4.0 phi units (Table 6). The sediments at all stations were poorly sorted, as the sorting coefficient is > 1.0 phi units. The OM contents of the sediments were uniform across the stations (Table 6), but with a distinctively higher percentage (25%) at LD2.

**Fig. 3:**
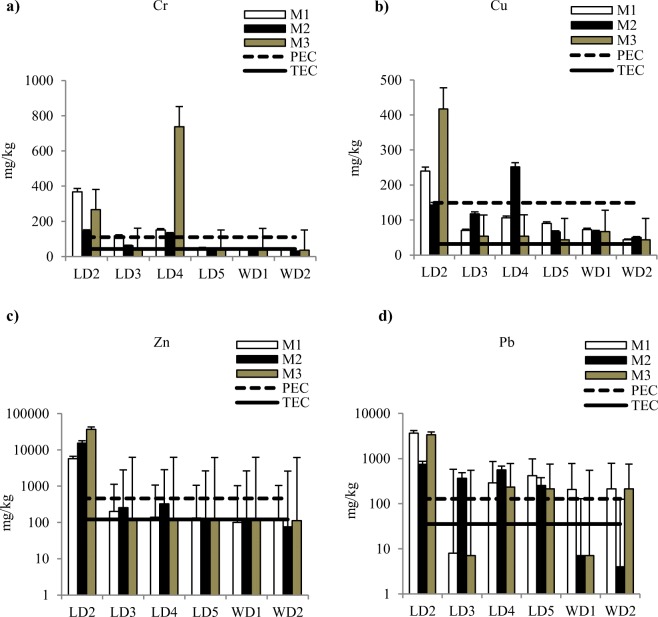
Mean metals contents in mg/kg (mean ± standard deviations, *n* = 3) in sediments samples collected from stations in Leyole and Worka rivers (Fig. 1c) in each of the three monitoring occasions M1–3. The different sediment quality guidelines are also presented here (TEC = Threshold effect concentration (mg/kg); Cr (43.4), Cu (31.6), Zn (121), Pb (35.8), (MacDonald et al. 2000b). PEC = Probable effect concentration (mg/kg); Cr (111), Cu (149), Zn (459), Pb (128), (MacDonald et al. 2000b)) (note the logarithmic scale for **c** and **d**)

The metal concentrations in the sediments varied across the stations in both rivers and also varied across the three monitoring occasions M1–M3 (Fig. 3). The maximum Cr concentration (740 mg/kg) was found at LD4, exceeding both the TEC and PEC guideline limits in all three occasions (Fig. 3a). Although the lowest sediment Cr concentrations were found at the two stations in the Worka river, the concentration was still close to the TEC guideline. Compared with the other Leyole stations, Cu concentrations were notably higher downstream of the steel factory at LD2, by at least a factor of 2 and 8 especially for the M1 and M3 monitoring occasions (Fig. 3b). At this station, the Cu concentrations exceeded the PEC in the three monitoring occasions. The lowest Cu concentrations were observed in stations of the Worka river, exceeding the TEC but not the PEC guidelines. As expected, the highest Zn concentrations were observed downstream of the steel factory (at LD2) in all monitoring occasions (M1–3) (Fig. 3c). In all occasions, the Zn concentrations exceeded the TEC and PEC guidelines. For both rivers, Pb concentrations exceeded the TEC and PEC guidelines (Fig. 3d).

**Fig. 4:**
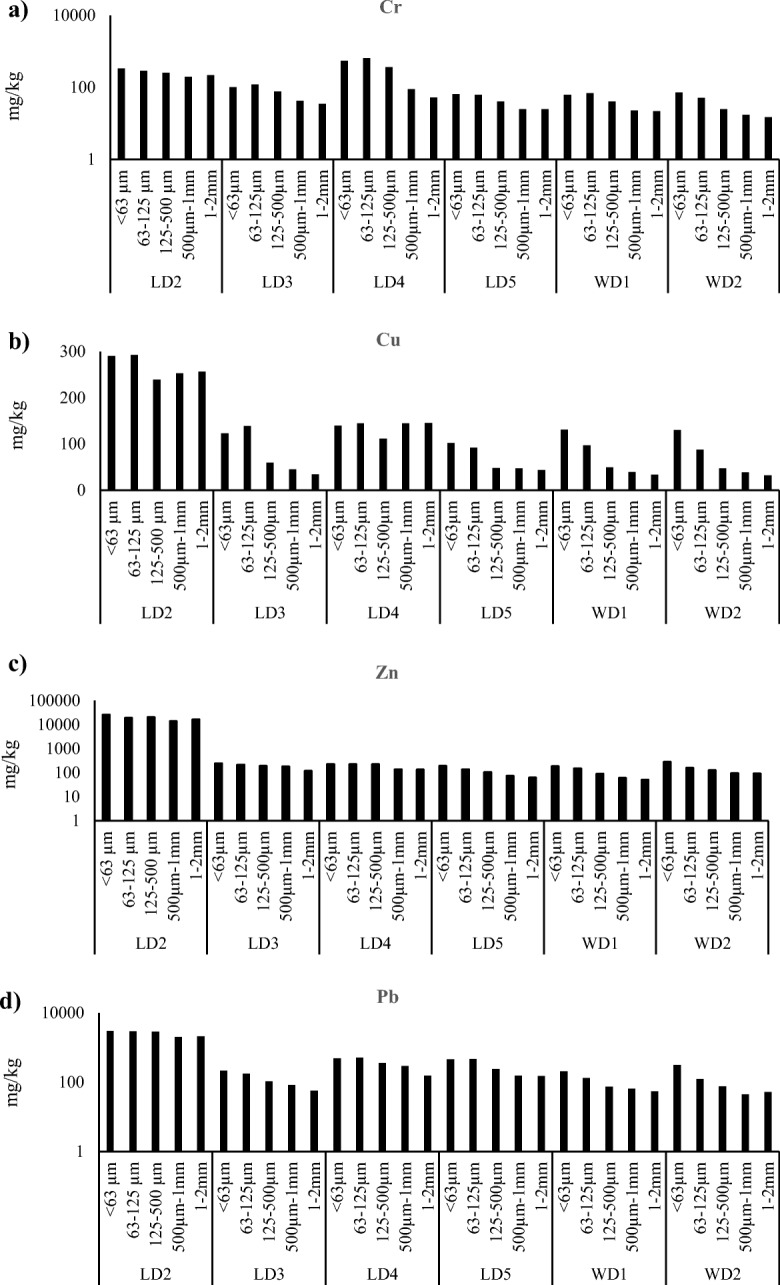
Metals contents (**a**–**d**: Cr, Cu, Zn, Pb) in mg/kg (mean ± standard errors; *n* = 3) in five grain size groups for sediment samples at the stations of the Leyole and Worka rivers (Fig. 1c), as average over three monitoring occasions M1-M3 (N.B. Cr, Zn, and Pb concentrations are in logarithmic scale)

The concentrations of metals in the Leyole and Worka river sediments generally showed a decreasing trend as grain size is increasing (Fig. 4). However, we also observed some considerable variation across sites. The Spearman rank correlation coefficients (Table 4) shows that significant (*p* ≤ 0.05) positive correlations between Cr and Pb, and Cu and Zn. Similarly, strongly significant (*p* ≤ 0.01) correlations were found between Pb and OM (Table 4).

**Table 4:** Spearman rank correlation matrix (*n* = 18) for the metal and organic matter contents, and sediment grain size fractions taken from six stations for three monitoring occasions; cf. Fig. 1

	Cr	Cu	Zn	Pb	OM	< 63 μm	63–125 μm	125–500 μm	500 μm–1 mm	1–2 mm
Cr		0.77	0.77	0.83*	0.91*	0.93*	0.89*	− 0.17	− 0.31	− 0.71
Cu			0.83*	0.60	0.74	0.75	0.54	− 0.61	− 0.49	− 0.71
Zn				0.83	0.85*	0.78	0.77	− 0.12	− 0.20	− 0.49
Pb					0.97**	0.93**	0.94**	0.06	− 0.37	− 0.66
OM						0.99**	0.91*	− 0.09	− 0.41	− 0.74
< 63 μm							0.87*	− 0.19	− 0.49	− 0.81
63–125 μm								0.12	− 0.26	− 0.60
125–500 μm									0.75	0.64
500 μm–1 mm										0.89*
1–2 mm										

*Significant at *p* ≤ 0.05, **at *p* ≤ 0.01

## Discussion

### Water and sediment quality in the Kombolcha rivers

Kombolcha catchments comprise steep uplands and gulley dissected flat landforms in a semi-arid climate. The flows of the rivers in the catchments are generally low and it is hard to conduct monitoring except in the wet season during which the hydrology flow of the rivers is relatively high. This study provides preliminary seasonal estimates of metal concentrations in the Leyole and Worka rivers and sediment over 2 years, 2013 and 2014 (Tables 1). The metal concentrations varied in the different sites of the rivers, and the concentrations of all metals were higher in the Leyole river compared with the Worka river. Similarly, large variations of metal concentrations were observed in the sediments of the rivers, likely because of differences in the sediment structure and grain size among samples (Håkanson and Jansson 1983).

**Table 5:** Model constants (*a*, *b*, and *c*) to estimate concentrations of metals in local standard and standard for standard (with 25 % fraction of < 63 μm and 10% OM) sediments in (mg/kg) for Cr, Co, Zn, and Pb

Metals	Constants		
	*a*	*b*	*c*
Cr	50	2	0
Cu	15	0.6	0.6
Zn	50	3	1.5
Pb	50	1	1

**Table 6:** Overview of sediment characteristics in the dark shaded columns in the left (for median grain size (phi units), sorting coefficients (phi units), fine grain size distribution (< 63 μm %), and OM (%)), and measured and corrected concentrations of Cr, Cu, Zn, and Pb in the left part columns; normalization is done with respect to a “standard sediment” with 25% fraction < 63 μm and 10% OM content

Stations	Median grain size (phi)	Sorting coefficient (phi)	% < 63 μm	% OM	Cr (mg/kg)		Cu (mg/kg)		Zn (mg/kg)		Pb (mg/kg)	
					Measured	Corrected	Measured	Corrected	Measured	Corrected	Measured	Corrected
LD1	2.8	1.4	36	25	335	275	290	172	25,790	18,469	2972	2678
LD2	0.3	1.4	23	25	101	105	123	116	247	271	216	273
LD3	3	2.1	8.7	6	550	816	139	174	230	371	487	739
LD4	0.5	2	6.9	7.2	65	101	103	138	194	340	457	727
LD5	3.2	2.4	6.3	6	63	101	131	188	187	348	209	345
WD1	− 0.5	3.3	4.2	4.2	73	125	130	200	284	583	315	543
WD2	2.8	2.6	36	3.8	335	275	290	172	25,790	18,469	2972	2678

The concentrations of Cr were notably elevated downstream of the tannery, particularly in 2014 (LD4, Fig. 2), in both water and sediments of the Leyole river. The increment in Cr concentrations is due to the outflows from the tannery factory (Zinabu et al. 2017a), and this is consistent with the findings of elevated Cr concentration downstream of the tannery is consistent elsewhere in Ethiopia (Gebrekidan et al. 2009) and sub-Saharan countries (see Table 7). Additionally, the high partition coefficient of the sediments for Cr (280 L/kg, which is estimated by Cr concentration in sediment, 740 mg/kg, divided by that in water, 2.66 mg/L) has likely contributed to the increased accumulation of Cr in sediments (Allison and Allison 2005).

**Table 7:** Maximum concentrations (mg/L) of selected metals in river water and sediments (mg/kg) reported in selected sub-Saharan countries and including the maximum concentrations of the metals in water and sediments from this study in Ethiopia (cf. Table 1)

Sub-Sahara countries	Cr		Cu		Zn		Pb		References
	Water	Sediment	Water	Sediment	Water	Sediment	Water	Sediment	
Egypt	0.06	185	0.05	333	0.12	743	0.02	95	Cu, Zn and Pb in water: (Abdel-Satar et al. 2017) Cr, Cu, Zn, and Pb in sediment: (El-Bouraie et al. 2010)
Ethiopia	25.9	738	2.45	417	3.31	36,612	0.98	3638	(cf. Table 1)
Ghana	177	5.06	–	–	8526	17.87	42.7	9.36	(Afum and Owusu 2016)
Nigeria	0.92	0.31	0.39	3.97	2.23	4.39	0.84	2.05	Metals in water: (Dan'azumi and Bichi 2010) Metals in sediments: (Sabo et al. 2013)
Tanzania	0.13	12.9	0.08	89.1	0.06	27.1	0.27	30.7	Metals in water: (Kihampa 2013) Metals in sediments: (Kishe and Machiwa 2003)
Uganda	0.02	103	6.3	78.3	3	351	3	90	(Fuhrimann et al. 2015)
Zimbabwe	2.48	16.1	0.23	38	0.50	100	1.02	41	For Cr: (Yabe et al. 2010) For Cu, Zn, and Pb: (Nyamangara et al. 2008)
Standard Limits	0.05	25	2	18.7	4	124	0.01	30.2	Water: (WHO 2011) Sediment: (USEPA 1997b)

The concentrations of Cu in the Leyole river has varied significantly both within each of the sampling period and between the two sampling periods (Table 2). This indicates the presence of infrequent discharges of Cu. The finding of higher Cu concentration in upstream parts of the Leyole river (LD2) compared with the other stations is possibly related to the outflows from the nearby steel processing factory, since Cu salts are commonly used in heating and galvanizing steel operation (Stigliani et al. 1993; Jumbe and Nandini 2009). Additionally, local open-pit and unlined landfills that are close to the river (at LD2) could be a source of Cu (Table 1), as solid wastes from the five factories are dumped there. Further study is needed to quantify metal pollution from the landfills.

The median concentrations of Zn were highest downstream of the steel processing factory outflow in the Leyole river (Table 1, Fig. 2c). For both water and sediments of the Leyole river, higher Zn concentrations were observed in 2014. This may be attributed to the larger-scale production of Zn galvanizing of steel products the factory (personal communication to Mr Ashebir Demeke 2015) and the legacy of effluent discharges from the zinc galvanizing process in previous years. The Zn concentrations varied significantly both within each of the sampling period and between sampling periods (Table 2), and like that of the Cu concentration, this suggests periodic discharges of Zn from a source.

The concentrations of Pb were relatively high in the downstream zone of the Leyole river, and this is likely due to the tannery outflows into the river (Zinabu et al. 2017a). Although the industrial sources in the upstream parts of the rivers were found to have low Pb emissions (Zinabu et al. 2017a), the relatively high Pb accumulation in the riverbed sediments (Fig.4) probably shows an incidence of Pb contamination from other sources. The finding of often relatively low concentration of the metal in water but high concentrations in the sediment (cf. Fig. 2 and 3) probably indicates a historical signal in sediments, while water results are more of a “snapshot view” (Chapman 1996).

All metals were observed to have maximum concentrations notably in the upstream part of the Leyole river (Table 1). While water sample handling, it is important to note that the water samples have been acidified before filtration of the suspended solids in the samples. This has possibly caused the release of adsorbed metals from suspended particles in the water samples. Although the river water has been observed to have relatively medium to high pH (Table 1), the sampling preparation method might be the main factor that led to the dissolution of suspended sediments and increasing the concentrations of metals. Furthermore, the steep uplands near the upstream of the rivers could drive runoff from the agriculturally dominated landscapes and overload sediments into the river water, and thus, the water samples might have contained considerable suspended solids.

The sediment grain size, which can be influenced by the runoff from the catchments, affects the metal contents of the sediments. Because of their larger specific surface area, the highest metal adsorption capacities can be expected for fine grains (or lutum) (< 63 μm) sediments (Wang 2000; Devesa-Rey et al. 2011). The correlations coefficients between the fraction < 63 μm and Pb, Cr, and Zn concentrations were strong for both the Leyole and Worka rivers (Table 4). However, the trend of decreasing metal concentration with increasing grain sizes was not straightforward, as we observed the opposite trend for all metal content especially for grain sizes > 63-μm fractions (see Fig. 4). A clearer trend would probably show further differentiation within the < 63-μm fraction (Devesa-Rey et al. 2011; Wang 2000). The organic matter (OM), which is most likely from upstream agricultural lands and industrial wastes, may have led to increased concentrations of Cr, Cu, Zn, and Pb in the sediments. The findings of a positive and strong correlation between the OM and Cr, Zn, and Pb metal concentrations support this view (Table 4). In a comparable river, Lin and Chen (1998) also reported higher Pb concentration with increasing OM.

To account for the effect of OM and grain size distributions on the concentrations of metals onto sediments, it is important to normalize the variation based on the above factors. Applying normalization procedures (i.e., used in the Netherlands) and comparing with a “standard sediment” (i.e., with 25% fraction of < 63 μm (i.e., lutum) and 10% OM content) yield results accounting for variations of metal content owing to all existing ranges of grain sizes and organic matter (Akele et al. 2016; Donze et al. 1990; Department of Soil Protection 1994). This procedure can be used to normalize legal threshold limits for the varying natural background concentrations of metals (Spijker 2012). In the procedure, a “formula^3^” is used to normalize the metal concentrations. The formula consists of metal concentrations both on local standard and standard for standard sediment lutum fraction and organic matter (i.e., 25% lutum and 10% OM)), and constants that are derived from eco-toxicological studies.

In this study, applying the above normalization procedures results in changes in the estimated toxicity effect of the sediment samples (Table 6). The number of stations exceeding the PEC limit increases from two to three for Cr; one to four for Cu; and one to two for Zn. Similarly, normalized Pb concentrations would increase at all stations.

In general, the industrial outflows is probably the main pollutant sources to affect the water quality and sediment quality of the rivers. Like that of Kombolcha industries, this problem is common across Ethiopia and many parts of Africa. It is reminiscent of the situation in Europe and the USA before the proliferation of licensing and regulation that arose from the 1960s to the 1970s in response to public concern and episodes of extreme pollution (Hildebrand 2002). To overcome the problems encountered in Ethiopia, it is important to understand the current situation of rivers and sediments and identify the needs and opportunities to build knowledge and capacity for better monitoring and management of rivers.

### Current status and improvement needed for monitoring of river water quality in Ethiopia

In Ethiopia, the attention given to the protection of river water and sediment quality. This is partly due to limited knowledge and information on pollutants. Of Ethiopia’s eight major river basins, only the Awash basin has any systematic monitoring (EMoWIE 2016), due to its relatively high economic significance compared with the other river basins. In the Awash basin, both the river and groundwaters have been monitored over the last 15 years (Personal communication to Ms. Konjit Mersha 2015). While the river water samples are collected monthly from 17 stations in the river including effluents from six beverage producers, the groundwater that is largely used for drinking water supply is monitored at the abstraction points. Both samples are analyzed only for hydro/chemical variables including total dissolved solids (TDS), dissolved oxygen (DO), biochemical and chemical oxygen demand (BOD, COD), electrical conductivity (EC), and pH. Although large commercial farms (e.g., for the international flower market) are reported to discharge a range of pollutants including metals, pesticides, and herbicides into the Awash River (Getu 2009; Endale 2011), the monitoring is restricted to the above conventional variables. No information is available regarding the state and trends in nutrients or pesticides emission and loadings into the river. Some sporadic, but extremely limited, monitoring of sediment, micro-pollutants, and metals have occurred; however, with poor laboratory facilities in the country, the processing of the samples for these chemicals is surely constrained (Table 8).

**Table 8:** Trends, needs, and current status of river monitoring in Ethiopia and improvement required for sustainable river basin water quality management

River water quality management		Regulatory status			
Trends required	Needs	Regulatory institution	Current status	Major constraint	Improvement needed
Monitoring	Knowledge of pollutants	• Federal environmental institutions and the Council (Ethiopian Ministry of Environment, Forest and Climate change) • Ethiopian Ministry of Water, Irrigation and Electricity • Regional and sectorial environmental institutions • Regional and sectorial water resources institutions • Ethiopian public health institution • Ethiopian water resources institute	• Local measurements • Conventional parameters • Monitoring of river water • Poor data availability	• Lack of appropriate instruments • Poor financial resources • Low technical capacities	• Monitoring of metals, river sediments, and special parameters (eco-toxicology, biomonitoring ) • Networking, remote sensing, and continuous measurements • Integrating effluents and river water monitoring • Addressing concerns in system complexity and methodology validity • Improved availability of data and efficient use of digital maps and telecommunication
	Agreed standards of quality		• General guidelines for all water uses ( based on WHO standards) • Limited local application of standards	• Poor financial resources • Limited monitoring networks and regulation • Insufficient interest on ecological protection	• Providing reliable data necessary to management information and transparent decisions • Development of guidelines for particular water use • Identifying and responding to violation of laws and regulations • Enhancing usefulness of research outputs
• Legislation and sustainable development	Relevant policy framework		• General rules and rigidity • Command and control • Polluter pay principles • Decisions by politicians and administration • Stick with standards borrowed from developed nations	• Poor enforcement • Confounding institutional settings and structures	• Decentralizing (developing local rules and flexibility) • Enforcement to regulation • Clearer structures and definition of roles and responsibilities in regulatory institutions • incentives to reduce the most important pollution problems • Public awareness and participation and enhanced communication to stakeholders • Commitment to international policies and setting out transboundary networks • Identifying difficulties in policy implementation and wider use of training programs

It is important to note that with the Ethiopian government policies to promote an industrial economy and concurrent growing of urbanization and intensive horticulture, adequate monitoring and understanding of the current situation and sources of all possible pollutants are needed. The Ethiopian government’s ambition to integrate Sustainable Development Goals (SDGs) into the Second Growth and Transformation Plan (GTP-II) cannot be effective without effective such as adaptable and low-cost monitoring and management regime. This is especially vital for encouraging socially responsible and environmentally safe sustainable manufacturing industries that are currently envisaged to be built through industrial parks across the country (FDRE 2016).

In Ethiopia, two federal institutions deal with water quality monitoring. The EMEFCC (Ethiopian Ministry of Environment, Forest and Climate Change) is the first institution that is responsible to establish national water quality criteria and pollution control policy (FDRE 2002; EEPA 2010). Accordingly, the EMEFCC has developed legally binding emission guidelines for eight categories of factories and is authorized to extend technical guidance to water bureaus and regional environmental institutions to regulate the emission (Zinabu et al. 2017a). The guidelines are generally prepared to protect ecological wellbeing of water resources, but not for other specifically designated water uses such as for irrigation and livestock water supplies. In the absence of such specific national guidelines, the application of international guidelines to protect water quality, like irrigation and livestock drinking water and protection of aquatic life, is recommended. The Ministry of Water, Irrigation and Electricity (EMoWIE) is the second institution which has a task of monitoring pollution of water resources and regulates in accordance with the Ethiopia Water Resources Management Regulation (No. 115/2005) (EMoWR 2004). The EMoWIE monitors water quality only for drinking waters and developmental projects inquiring specific information on water quality. Both the EMoWIE and the EMEFCC has established water quality monitoring networks only in the Awash basin (Benoist 2002). While both EMoWIE and EMEFCC are working for a common purpose, the lack of a clear definition of roles and responsibilities between the institutions prevents coordination and effective use of information (Table 8).

Based on our assessment of the institutions, limited financial sources hinder acquisition of the necessary field and laboratory equipment, and human capacity is insufficient to deal with current water quality assessment needs of the above institutions. Additionally, the absence of adequate monitoring infrastructures and aging of the existing ones are obstacles to establishing effective monitoring networks. It is clear that developing the capacity and financial needs of monitoring are vital to reverse the current trends of water quality degradation (Table 8) and there is a need to focus on adaptable and affordable approaches for water quality monitoring.

In Ethiopia, water quality standards for river waters merely follow the WHO guidelines for drinking water (EMoWR 2004). While current economic development of Ethiopia is moving at a fast pace, overall environmental protection is still limited, and local application of water quality guidelines is absent (Damtie and Bayou 2008; Zinabu et al. 2018). The WHO guidelines outlined all the physicochemical and bacteriological components that need to be monitored in waters (Alemu et al. 2015). However, in Ethiopia, due to the limited capacity and the lack of adequate laboratory instruments, micropollutants such as metals are commonly left out in the water sample analyses (EMoWIE 2016; Zinabu et al. 2018). The WHO guidelines are not designed to assess ecosystem health, and many of the toxic substances included by the WHO have no legally mandated monitoring in Ethiopia (WHO 2011). In addition to the limited monitoring networks and regulation, low levels of financing for environmental research and monitoring has hindered policy making especially in providing reliable water quality information and capacity to develop national water quality guidelines (Awoke et al. 2016). The environmental regulation is based on the principle of “polluter pays,” but enforcement by regional and local water bureaus and environmental institutions is poor because of the fundamental lack of capacity in the institutions (EMoWIE; 2016).

Many of the sub-Saharan countries adopted water quality guidelines either from developed countries or international guidelines. However, this approach often does not consider ongoing economic, social, and technical needs (Ongley 1993) and is hard to take as appropriate guidelines for the nations. Developing a new water quality paradigm is important and this can be achieved through prioritizing development of institutional capacity so as to generate reliable water quality information and efficiently formulate practicable guidelines. For instance, Ghana and Kenya use international guidelines to assist the development of national water standards. They used their own water quality data to derive national drinking water quality standards based on the WHO guidelines (Peletz et al. 2016). Alternatively, it is possible to adopt another country’s guidelines. For example, in Nigeria, the Federal Environmental Protection Agency (FEPA) reviewed the water quality standards from Australia, Brazil, Canada, India, Tanzania, and the USA to derive their own Interim National Water Quality Guidelines and Standards, including those for aquatic life protection and irrigation and livestock watering (Ute et al. 1997).

Several studies report that the metal concentrations in rivers pose high risks in many sub-Saharan countries (see Table 7). However, like Ethiopia, many of the sub-Saharan countries have not developed national water and sediment quality standards. With increasing uncontrolled waste discharges and anthropogenic inputs into sub-Saharan rivers, the problem of metal pollution is a major concern (Peletz et al. 2018; Abdel-Satar et al. 2017). The problem is critical as more river waters are intensively used for irrigation and storage and the dilution capacity of the rivers for pollutants is diminishing (Abdel-Satar et al. 2017). Monitoring of metals and understanding of their impact on river water quality are therefore a pressing need in the regions. This requires prioritizing rivers and sediment quality problems in guiding water safety management and ensuring environmental health through a policy framework that sets effective and practicable monitoring and regulation of metals in rivers and sediments (Table 8).

### Monitoring in transboundary rivers and lakes of Ethiopia

There are seven major transboundary rivers in Ethiopia. With Ethiopia’s commitment to the SDGs, it is important to establish monitoring networks for the rivers that cross to the neighboring countries. Currently, Ethiopia is a member of the Nile Basin Initiatives (NBI) (Nile Basin Initiative 2016), which is an intergovernmental partnership of ten Nile riparian countries (http://nileis.nilebasin.org/content/nile-basin-water-resources-atlas), and endorsed regional monitoring networks, including for water quality. Across the region, the monitoring still remains at an early stage and data exchange among stations and central data repositories are poor (Abdel-Satar et al. 2017; Nile Basin Initiative 2016). The recent NBI development in designing regional hydrometric system to enhance existing networks, such as IGAD-HYCOS Program based on national needs and limitations, is an important step in improving the monitoring system for the river (Nile Basin Initiative 2016). The monitoring is still based on meteorological measuring of daily rainfall and temperature, and hydrometric measuring of rivers or lake water levels. The Nile river is increasingly used for irrigation and energy generation (Abdel-Satar et al. 2017); thus, it is time to monitor water quality, sediment transport, and groundwater quality in the basin. This needs immediate attention, as the current situation likely poses pollution risk and affects the ecological health of the rivers.

It is important to mention that the NBI comprises three of the major transboundary rivers of Ethiopia (viz. the Blue Nile, Tekezze, and Baro rivers). Three of the transboundary Rivers (viz. the Omo, Shebelle, and Mereb rivers) are not included in the NBI and flow across the boundaries with Kenya, Somalia, and Sudan, respectively. There is no clear international collaboration of monitoring networks for these rivers. Additionally, Ethiopia has twelve big lakes. Apart from the Lake Tana, which is the source of the Blue Nile River and included in the NBI, most of the other lakes are found in the rift valley of the country. Population pressure and rising economic interest have increased pressures on the lake waters (Francis and Lowe 2015). For instance, Larissa et al. (2013) report high metal concentrations both in the water and tissue of edible fish species living in the Koka and Awassa lakes, due to uncontrolled discharges of industrial effluents into the lakes. More attention is also needed to establish domestic monitoring networks to the lakes of the Ethiopian rift valley and this can help in preparing proper policy actions at local and national levels.

## Conclusion and the way forward

In Ethiopia, river monitoring is not extensive enough to provide information necessary for water and sediment quality management. This is a preliminary study that demonstrates the management of metal pollution is a serious issue in Kombolcha, a city that illustrates the same problems across Ethiopia and, generally, in the sub-Saharan countries. To understand the causes and sources of metal pollution in the Kombolcha rivers. Additional studies are needed to understand possible pollution sources in the city, especially from industrial discharges, agricultural activities, catchment geology, and local landfill emissions. These studies also need to cover the sources’ characteristics over time and assess their historical patterns. To ensure effective monitoring of rivers and sediments and provision of monitoring information in Ethiopia, commitments from jurisdictions is urgently needed in developing institutional capacity and implementing adaptable monitoring techniques.

In Ethiopia, there is a clear need for compliance with water protection requirements and feasible guidelines responsive to the environmental problems at hand. This could be done either by reviewing the applicability of international or comparable countries’ water quality guidelines. Like that of Ghana and Kenya, building a monitoring network that provides baseline data to inform national policy is an alternative measure. Similarly, like in Nigeria, reviewing the process that includes consultation with a range of interested parties, for example, from the private sectors, higher education institutions, non-governmental organization, and the interested public, can be another option (Ute et al. 1997).

Low-cost techniques and advocacy for public and private partnership can help to overcome the limited institutional structures, lack of adequate instruments, and deficiencies in necessary skills for monitoring rivers and sediments. The growing use of information technology using smartphones provides an example of new opportunities that can both transmit monitoring data and raise local awareness to water quality monitoring. For instance, a mobile phone application implemented to facilitate water quality data collection within the national public health agency is recommendable for effective intervention (Emily et al. 2015). The information acquired from smartphones can readily be linked to GIS and remote sensing techniques and support modelling of river water quality and monitoring of rivers especially in relatively large basin areas (Dube et al. 2015; Ritchie et al. 2003). If coupled with modelling of pollutant, information on emissions from point and non-points sources provides improved risk assessment of pollutants in rivers (Brack 2015; Daughton 2014; Zinabu et al. 2017b).

Cost-effective monitoring networks like engaging citizens in science, with the EU-funded Ground Truth project (http://gt20.eu/), provide useful data at low cost, as well as stimulate citizen interest in water quality issues (Katsriku et al. 2015). This can be useful for Ethiopia to strengthen local institutional capacity even in remote and rural areas. Furthermore, simple monitoring techniques such as mini stream assessment scoring system applied in South Africa is a feasible option to manage water and sediment quality information at local scales (www.groundtruth.co.za/projects/minisass.html) (Aceves-Bueno et al. 2015).

## Footnotes

1 Equivalent with “standard deviation,” $\mathrm{S}.\mathrm{C}=\Big(\frac{1}{2}\left(\mathrm{grain}\ \mathrm{siz}<84{\%}_{\mathrm{of}\ \mathrm{total}}\right)-\left(\mathrm{grain}\ \mathrm{size}>16{\%}_{\mathrm{of}\ \mathrm{total}}\right)$ Sediments with S.C. < 1.0 phi-units are considered as “well sorted”; > 1.0 phi units as "poorly sorted" (Håkanson and Jansson 1983)

2 phi unit =  − log_2_grain size; e.g., for grain size = 0.63 = 2^−4^ mm; phi size=4.0 phi units

3 $$ {C}_{\mathrm{normalized}\ \mathrm{local}\ \mathrm{metal}}={C}_{\mathrm{local}\ \mathrm{metal}\mathrm{s}}\times \frac{a+\left(b\times \mathrm{lutum}\right)+\left(c\times \mathrm{organic}\ \mathrm{matter}\right)}{a+\left(b\times 25\right)+\left(c\times 10\right)} $$, in which:

- *C*_normalized local metal_ are corrected/normalized metal concentrations (mg/kg) on local/sample sediments
- *C*_local metal_ are concentrations (mg/kg) of metal on local/sample sediments
- *a*, *b*, *c* are constants depending on the elements as given in Table 5
- lutum locally measured lutum fraction as percentage of dry weight
- organic matter is locally measured organic matter content as percentage of dry weight
